# Retraction: Fingolimod inhibits proliferation and epithelial- mesenchymal transition in sacral chordoma by inactivating IL-6/STAT3 signaling

**DOI:** 10.1042/BSR-20200221_RET

**Published:** 2020-07-03

**Keywords:** epithelial-mesenchymal transition, FTY720, IL-6/STAT3 signaling, proliferation, sacral chordoma

This Retraction follows an Expression of Concern relating to this article previously published by Portland Press.

This article is being retracted from *Bioscience Reports* at the request of the authors following receipt of a notification from a reader, alerting the authors to duplications in the E-cadherin and Vimentin western blots in Figure 2C.

The authors have been unable to replicate the results and wish to retract the article. The Editor-in-Chief and Editorial Board agree with the retraction.

